# Timeline of History of Hypertension Treatment

**DOI:** 10.3389/fcvm.2016.00003

**Published:** 2016-02-23

**Authors:** Mohammad G. Saklayen, Neeraj V. Deshpande

**Affiliations:** ^1^V.A. Medical Center, Wright State University Boonshoft School of Medicine, Dayton, OH, USA; ^2^Ohio State University, Columbus, OH, USA

**Keywords:** hypertension, randomized controlled trials, history, treatment outcome, multicenter trial

## Abstract

It is surprising that only about 50 years ago hypertension was considered an essential malady and not a treatable condition. Introduction of thiazide diuretics in late 50s made some headway in successful treatment of hypertension and ambitious multicenter VA co-operative study (phase 1 and 2) started in 1964 for diastolic hypertension ranging between 90 and 129 mmHg and completed by 1971 established for the first time that treating diastolic hypertension reduced CV events such as stroke and heart failure and improved mortality. In the following decade, these results were confirmed for the wider US and non-US population, including women and goal-oriented BP treatment to diastolic 90 became the standard therapy recommendation. But isolated systolic hypertension (accounting for two-thirds of the 70 million hypertensive population in USA alone) was not considered treatable until 1991 when SHEP study (systolic hypertension in elderly program) was completed and showed tremendous benefits of treating systolic BP over 160 mmHg using only a simple regimen using small dose chlorthalidone with addition of atenolol if needed. In the next two decades, ALLHAT and other studies examined the comparability of outcomes with use of different classes and combinations of antihypertensive drugs. Although diastolic BP goal was established as 90 in the late 70s and later confirmed by HOT study, the goal BP for systolic hypertension was not settled until very recently with completion of SPRINT study. ACCORD study showed no significant difference in outcome with sys 140 vs. 120 in diabetics. But recently completed SPRINT study with somewhat similar protocol as in ACCORD but in non-diabetic showed almost one-quarter reduction in all-cause mortality and one-third reduction of CV events with systolic BP goal 120.

Over the last several decades, the term “essential hypertension” has become entrenched in our medical vocabulary. We not only use it in our usual medical lingo but the term was actually codified in ICD-9. As a medical community, we do not categorize any other common pathological conditions, such as obesity, NIDDM, or CAD as “essential.”

Moreover, in order to distinguish the common variety of hypertension from a specific etiology-related hypertension – so-called secondary hypertension – the most logical alternate term would have been “primary” hypertension. But why and how did the term “essential hypertension” come about? To find the answer, one needs to delve into the history of the pharmacological treatment of hypertension.

The history of hypertension goes back a long way (1). In ancient Chinese and Indian Ayurvedic medicine, the quality of an individual’s pulse, as felt by gentle palpation by the trained physician, was a window into the condition of the cardiovascular system. What was called “hard pulse” possibly would qualify for the modern term of hypertension. Any article on the history of hypertension, however, is incomplete without a mention of Akbar Mahomed’s contribution in developing the modern concept of hypertension. In the late nineteenth century, Frederick Akbar Mahomed (1849–1884), an Irish-Indian physician working at Guy’s hospital in London, first described conditions that later came to be known as “essential hypertension,” separating it from the similar vascular changes seen in chronic glomerulonephritis such as Bright’s disease. Some of the noteworthy contributions of Akbar Mahomed were the demonstration that high BP could exist in apparently healthy individuals, that high BP was more likely in older populations, and that the heart, kidneys, and brain could be affected by high arterial tension (Interested readers may read about Akbar’s life in a detailed account written by Cameron in Kidney international) (2, 3). However, only with the advent of the mercury sphygmomanometer in the early twentieth century and defining of the systolic and diastolic BP by appearance/disappearance of Korotkoff sounds as heard *via* the stethoscope, the modern quantitative concept of hypertension – broken into systolic and diastolic categories – came into existence. By the middle of the twentieth century, checking BP by sphygmomanometer became part of the routine physical examination in hospitals and clinics (4).

Hypertension, however, was not always considered a disease as we know it now. President Franklin D. Roosevelt was given a clean bill of health by his physician even when his BP was recorded as ~220/120. A few years later while at Yalta, Winston Churchill’s personal physician noted in his diary that President Roosevelt “appeared to be have had signs of ‘hardening of the arteries disease’ and had a few months to live.” Subsequent events demonstrated the truth of his diagnosis. President Roosevelt ultimately had a fatal hemorrhagic stroke 2 months later, and his death brought hypertension’s potential as a deadly malady to the lime light (5).

Three years after Roosevelt’s death, the pivotal National Heart Act was signed into law by President Truman. The Act created the path for the study of heart diseases and resulted in several studies including the Framingham Heart Study. The Framingham studies consistently showed that hypertension, such as hyperlipidemia, was associated with many cardiovascular morbidities such as stroke, heart failure, and heart attacks leading to premature deaths and the risk was clearly higher with higher blood pressure (systolic and diastolic). Even before the Framingham studies, many insurance companies had already begun measuring BP for policyholders’ physical examinations. Furthermore, several studies done by the Actuarial Society of America pointed toward the higher morbidity and mortality associated with higher BP (6, 7).

Yet, throughout the 1960s, the debate continued in the medical community regarding whether a need existed for treating the common variety of hypertension, by then aptly named “essential hypertension” because it was deemed an unavoidable, hence essential, component of the aging process. Attempts to treat hypertension, with the few drugs that were available at the time, often caused more misery and earlier demise for the patients than leaving them untreated. The prevailing attitude in the academic community was expressed in an editorial in the Archive of Internal medicine in 1965 by two professors of medicine from New York.

To quote

It is common experience that many patients live medically uneventful lives in spite of prolonged and considerable blood pressure elevation. In our study group’s experience with 241 living and continuously employed hypertensive patients, followed from 10–25 years, a so called benign course was the rule, not the exception … A drug that will maintain BP in the normal range in the supine as well as upright position without adverse physiological effects for all 24 hours over a period of years, when and if available, may well make medical history …

One needs only to look back at the past 50 years to be amazed and deeply concerned at the worldwide enthusiasm generated by many proposed therapies for hypertension which eventually met their deserved doom – oblivion…

Acceptable techniques for obtaining the necessary proof are presently not available. We believe that critical techniques designed for a more precise and scientific answer to the problem under discussion will appear much sooner in an atmosphere of less enthusiasm and more caution in interpreting the results and implication of this form of therapy (8).

For more details on this controversy, interested readers are referred to Dr. Moser’s review on the early years of hypertension treatment (9).

Some reconciliation was therefore necessary between the two opposing views: is the common variety of hypertension, or essential hypertension, a benign condition or is it a killer disease? Serendipitously, as it happened for many other important breakthroughs in science, a few lucky developments occurred around the same time and helped in resolving the ongoing controversy.

1. The first Randomized placebo-Controlled clinical Trial (RCT) in the history of medicine was conducted by Medical Research Council (MRC of UK) in 1948 (studying the effectiveness of streptomycin for the treatment of tuberculosis). This provided the tool for scientific proof of efficacy of any treatment (10).
2. Thiazides and thiazide-like compounds were developed from the sulpha molecule and they were found to have natriuretic properties. First to come was chlorothiazide, followed by hydrochlorothiazide, and then chlorthalidone a few years later. These drugs showed potent hypotensive effects. Small studies demonstrated thiazides’ tolerability and effectiveness of this new group of drugs in lowering BP (11).
3. Arthur Guyton, the famous renal physiologist, developed his theory of pressure natriuresis and hypothesized that abnormal pressure natriuresis curves can explain all types of hypertension (12).
4. The Veterans Administration Medical Centers – a chain of federal hospitals dedicated to the care of war veterans – added to their missions of clinical care and medical education the lofty goals of pursuing medical research. Thus, the VA Co-operative research program was started. Having the VA hospital system with many hospitals, scattered across USA, under one authority, made it possible for large multi-center clinical studies to be carried out quickly and with relative ease (13).

All of these lucky developments eventually produced the first multi-center hypertension treatment trial (the first VA Co-operative research study on hypertension), which was published in JAMA in 1967. The major findings of these and other landmark studies that followed in the subsequent 50 years are highlighted in this timeline. Considering the importance of the topic, there had been very few attempts to detail the history of hypertension treatment. Noteworthy are Dr. Moser’s personal account and a more detailed timeline created by Theodore Kotchen (14).

In creating this timeline, I opted to choose studies that were landmark in nature and answered a hitherto unresolved question regarding management of “essential hypertension.” Subsequent studies that reaffirmed these answers or only elaborated on the main theme were mostly omitted to keep the timeline short and readable. For each selected study, we gave the main hypothesis of the study, brief outline of the study methodology and the main result(s) along with a brief commentary as to the historical importance of the study. I have avoided meta-analyses, however, important they may be. We also avoided basic science studies and I have purposefully left out newer experimental therapies like renal sympathetic ablation therapy for resistant hypertension, since it is still evolving.

Following are the brief descriptions of the studies that make up the timeline of this review (Figure 1; Table 1).

**Figure 1:**
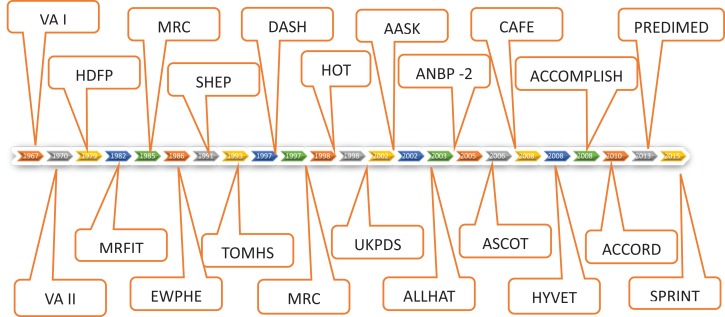
**VA-co-op study phase 1 followed by 2 established for the first time that diastolic HTN > 90 to 129 was treatable with available drugs and reduced stroke, CHF, and mortality**. HDFP study affirmed that BP treatment target to diastolic goal of 90 gave much better CV outcome results than usual BP treatment. MRC and EWHPE confirmed this for younger and older patients, respectively, in non-US population. MRFIT study showed that of three risk factors for CHD (hyperlipidemia, smoking, and hypertension) only hypertension was effectively treatable by drugs available that time. SHEP study broadened the definition of treatable hypertension to include isolated systolic hypertension, treatment of which in elderly gave profound CV and mortality benefits. DASH study convincingly showed the benefits of Mediterranean type diet in lowering BP and that salt restriction adds to that benefit. HOT study established that lowering diastolic BP goal <90 (85 or 80) does not add any further benefits. TOMHS and MRC 2 were relatively minor studies. UKPDS Hypertension studies showed that moderately tight BP control <150/85 goal-reduced diabetic mortality by 32% – much higher level of benefits than in non-diabetics. AASK study was done in African-Americans with CKD and showed that tight BP control over usual BP control did not affect CKD progression but use of ACEI caused superior reno-protection over CCB. ALLHAT study showed that use of thiazide drugs (Chlorthalidone) did not increase incidence of MI or mortality over other classes of drugs (CCB, ACEI, or AB). It also showed incidence of CHF was more with use of AB, CCB, and ACEI than CTDN. ASCOT study showed superiority of combination of ACEI and CCB over BB and thiazide (HCTZ) in preventing CV outcomes. CAFE, a sub study of ASCOT showed that BB failed to lower central aortic BP as opposed to peripheral BP. HYVET showed that treatment of hypertension in very elderly (>80) is even more beneficial than in any other age group. ACCOMPLISH study showed superiority of combination of ACEI and CCB over ACE and thiazide (HCTZ and not CTDN) for CV outcomes. ACCORD study showed that in diabetics, lowering BP target to 120 sys over conventional 140 added no further reduction in CV or renal outcomes. SPRINT study showed significant mortality and cardiovascular benefits in group with Systolic BP treatment goal of 120 compared to goal of 140 in non-diabetic patients.

**Table 1:** **Time line of hypertension**.

Study	Year	Primary question/issues	Conclusion of the study/impact
VA-1st	1967	Is severe hypertension (dias) 115–129 treatable	Yes, less stroke/CHF
VA-2nd	1970	Same question for moderate BP (90–115)	Treated group less stroke/CHF
HDFP	1979	Goal-oriented BP therapy better than usual therapy?	Yes. Targeting BP goal of dias 90 reduced CVA by 36% more
MRFIT	1982	Lowering BP and lipid and stopping smoking may reduce CHD mortality	No difference in CHD mortality 17.9 vs. 19.3% (per 1000)
MRC	1985	Hypertension treatment in younger patients (35–64) is beneficial also?	Yes. Total CV events 286 in treated group vs. 352 in control (*p* < 0.05)
EWHPE	1986	Hypertension treatment in exclusively older people (60) beneficial?	Yes. Mortality reduction 26% decrease in CV mortality 43%
SHEP	1991	Is treatment of systolic hypertension beneficial	Treating isolated systolic hypertension over 160 prevented stroke (ARR 3%), MI, and all CVD
TOMHS	1993	Outcome of 5 different classes BP meds vs. placebo	BP lowering similar among all classes CV events and death reduced (ARR 2.2%)
DASH	1997	Does Mediterranean diet with or without salt restriction lowers BP?	Compared to western diet it lowers bp and salt restriction adds to the effect
MRC	1997	Salt reduction in older people Lowers BP?	Reducing salt intake to 2 g Na lowered BP 7.2/3.2 mmHg
HOT	1998	Lowering Dias BP to 85 or 80 beneficial compared to standard 90 goal	No significant benefit in whole study but small benefit in diabetic
UKPDS	1998	Multiple studies 2 involved BP Tight BP control and agents (captopril vs. atenolol)	Group target <150/85 had 32, 44, and 34% less death, stroke, and retinopathy, respectively. No difference in ACEI group vs. BB
AASK	2002	To reduce progression of CKD BP goal mean 92 better than 105 ACEI, BB, or CCB better as drug?	No difference in mean BP goal of 92 vs. 105. ACEI use protected progression of CKD better than CCB
ALLHAT	2002	Compared to old thiazide (CTDN) new class of BP drugs CCB, ACEI, or AB has better outcome? AB gr closed for high incidence of CHF	No difference in MI, mortality, or CKD progression among 3 classes. CTDN vs. CCB for CHF RR 1.38. CTDN vs. ACEI for stroke and CHF RR 1.15 and 1.19
ANBP2	2003	ACEI vs. thiazide (HCTZ) for CV outcomes in Australian	In this study unlike ALLHAT, ACE was better all CV events RR was 0.88
ASCOT	2005	CCB and ACE inhibitor compared to BB and thiazide for BP control	CCB and ace inhibitor combination group showed better CV outcomes
CAFÉ	2006	Why Betablocker for BP does not prevent stroke	Betablocker lowers peripheral BP but not central (aortic) BP
HYVET	2008	Should we treat elderly (>80) hypertensive (sys > 160)	Yes. Treated group had 30% less stroke and 64% less CHF, 21% less death
AC-SH	2008	Combination of ACEI + CCB better than ACEI + thiazide (HCTZ)	ACEI + CCB group had 2.2% ARR of composite CV events and death
ACCRD	2010	In diabetics Goal BP sys < 120 better than 140?	No significant difference in mortality, total CV events, or renal protection
SPRINT	2015	Same as ACCORD but in non-diabetic	27% improved all-cause mortality and 25% improvement in primary CV outcomes

## Timeline of Key Hypertension Studies

VA cooperative study phase 1 – 1967 VA co-operative study on HTN. First Randomized controlled trial of hypertension treatment anywhere in the world. Truly ground breaking study.

The Veterans Administration (VA) cooperative study on antihypertensive agents was a major milestone achieved in medicine. This was the first adequately powered placebo-controlled, RCT of antihypertensive therapy (15). The phase 1 of the study examined active treatment (hydrochlorothiazide, reserpine, and hydralazine) vs. placebo in 143 veterans with severe hypertension (diastolic blood pressure 115–129 mmHg) and achieved an average fall of blood pressure by 43/30 mmHg in the treated group. The recruitment started in 1964 and average follow-up was about 1.5 years. The results of the study showed clear morbidity and mortality benefit, most remarkably in reduction in progression to accelerated/malignant hypertension in the treatment group.

Study summary:

- Total patients – 70 in each group
- BP achieved – 91.6 vs. 119.7
- Events-
  – Death: 4 in control, 0 in treatment group
  – Morbid Events: 27 in control, 2 in Rx group

Morbid events included progression to malignant hypertension as evidenced by fundal hemorrhage, CVA, MI, heart failure, and dissecting aneurysm.

VA cooperative study phase 2 – 1970 This phase of the study examined the benefit of active treatment vs. placebo in moderately severe hypertension (diastolic 90–115).

Recruitment for this phase started same time in 1964 as with the first phase group but being less severe disease, and this study needed larger sample size and longer follow-up. They were followed for a longer duration (mean 3.8 years). Using the same active treatment as in the first study, this study achieved an average fall in diastolic BP by 19 mmHg in the treatment group. The results showed significant mortality and morbidity benefits (16).

Study summary:

- Inclusion – diastolic BP > 90 < 115
- Total number – 380 all males (186 vs. 194)
- Follow-up – average 3.9 years
- Mean age – 52.0
- Rx – HCTZ, reserpine, hydralazine
- Initial BP – 165/105 vs. 162/104
- Drop out – 15% equally divided
- BP achieved: 138/91 vs. 166/105
- BP dropped average (treatment vs. control)
  - 27.2 vs. 4.2 (+) systolic
  −17.4 vs. 1.2 (+) diastolic
- Deaths: 19 vs. 8
- Total morbid events: 56 vs. 22 (29 vs. 12%)
- CVA: 20 vs. 8
- CHF: 11 vs. 0
- Malignant HTN: 4 vs. 0
- CAD: 13 vs. 11

Hypertension Detection and Follow-Up (HDFP) 1979 First study to demonstrate benefit in mortality and morbidity by aggressive, goal directed blood pressure treatment with stepwise incremental therapy as opposed to more casual BP management without trying to reach a target BP. This study set the ground rules for future management of BP using incremental therapy – a new concept in managing chronic diseases.

Published in 1979, the Hypertension Detection and Follow-Up (HDFP) trial was another land mark trial of antihypertensive therapy and was the first study to demonstrate a mortality benefit of goal-directed, stepped care blood pressure treatment compared to usual care (17). The study established the practice of stepped care approach to achieve BP goal which became the norm of hypertension treatment strategy ever since. Of note, this study exclusively used chlorthalidone (CTDN) as opposed to HCTZ and all subsequent NIH sponsored studies did the same.

Study summary:

- Total number: 5485 (Stepped care) vs. 5455 (Usual Care)
- Inclusion: diastolic BP > 90 mm
- Male:Female – 50:40
- Intervention: stepped care vs. usual care
- Drugs: chlorthalidone, reserpine, K-sparing diuretics, methyldopa, hydralazine, guanethidine
- Entry BP: 159/101 vs. 158/101
- BP at goal first year: 51.8 vs. 29.4%
- BP at goal fifth year: 64.9 vs. 43.6%
- Mortality: (6.4 vs. 7.7%) RRR-17%
- CVA: 102 (1.8%) vs. 158 (2.8%) RRR 36%
- CHD: 171 (3.1%) vs. 189 (3.5%)
- Total CV events: 273 (4.9%) vs. 347 (6.4%)

Multiple Risk factor Intervention (MRFIT) 1982 Not a true antihypertension study per se but BP reduction was one of the 3 risk factors treated.

The Multiple Risk Factor Intervention Trial (18) was an ambitious randomized primary prevention trial to test the efficacy of a multifactor intervention program on mortality from coronary heart disease (CHD) in 12,666 high-risk men aged 35–57 years. Men were randomly assigned either to a special intervention (SI) program consisting of stepped-care treatment for hypertension, counseling for cigarette smoking, and dietary advice for lowering blood cholesterol levels, or to their usual sources of health care in the community.

• Age: 35–37 years
• Study randomization: 12,666
  6428 – Intervention (SI)
  6438 – Control (UC)
Intervention:
–Dietary counseling
–Smoking counseling
–Treatment of HTN
–Lower cholesterol
  - Duration: average 7 years
  - Mortality from CHD: 17.9 vs. 19.3 (per 1000)
  - Total mortality: 41.2 vs. 40.4 (per 1000)

None of the difference is significant *p* > 0.5.

The study results played an important role in the direction of hypertension treatment in the ensuing decade. The failure of the study to achieve reduction in mortality in the intervention group was blamed on hypokalemia in some of the thiazide-treated patients and newer antihypertensive drugs were promoted by pharmaceutical industry over thiazide and thiazide such as diuretics like Chlorthalidone. On retrospect, the real cause for failure was that the study was planned prematurely. There was no Statin yet and arrival of Statin in the 90s made all the difference in CAD mortality.

MRC trial of treatment of mild hypertension: ­principal results 1985 First definitive study to provide evidence of benefits in treating mild hypertension in younger cohort – men and women aged 35–64 years.

The primary objective of the trial (19) was to study the effect of the drug treatment of mild hypertension on the rates of stroke, of death due to hypertension, and of coronary events in men and women aged 35–64 years. The secondary objectives of the trial were to compare the effectiveness and adverse effects of two antihypertensive drugs bendrofluzide and propranolol.

Study Summary:

Design: single blind RCT based almost entirely in general practices
No of participants: 17,354
Active treatment (bendrofluzide or propranolol): 8700
Placebo: 8654
Duration: 5 years
Stroke: active treatment (60) vs. placebo (109), *p* < 0.01
Coronary events: active treatment (222) vs. placebo (234)
All cardiovascular events: active treatment (286) vs. placebo (352), *p* < 0.05
Mortality from all causes: active treatment (248) vs. placebo (253)

The authors concluded that to prevent one stroke 850 individuals would have to receive treatment for a year.

European Working Party High Blood pressure in the Elderly (EWHPE) 1986 First major study of hypertension in exclusively elderly population

For the EWHPE trial (20, 21), 840 men and women over 60 years old, with a systolic blood pressure in the range 160–239 mmHg and a diastolic pressure in the range 90–119 mmHg, were randomized to receive active treatment (hydrochlorothiazide with triamterene) or matching placebo.

Study Summary: overall

- A non-significant change in total mortality rate (−9%).
- Reduction in cardiovascular mortality (−27%, *p* = 0.037)
- Decrease in cardiac mortality (−38%, *p* = 0.036) and an insignificant
- Decrease in cerebrovascular mortality (−32%, *p* = 0.16).

Study results double-blind part of the trial:

- Decrease in total mortality rate (−26%, *p* = 0.077).
- Decrease in cerebrovascular mortality on treatment (−43%, *p* = 0.15).
- Decrease in cardiac deaths (−47%, *p* = 0.048).
- Decrease in deaths from myocardial infarction (−60%, *p* = 0.043).

Systolic Hypertension in the Elderly Program (SHEP) 1991 First randomized controlled trial to show benefits of ­treating isolated systolic hypertension which prior to this most ­remarkable study was considered benign or not amenable to treatment, though isolated systolic hypertension showed more associations with CV morbidities and ­mortalities per Framingham and other studies. The success of this study opened up the ­treatment option to 50 million people in US alone.

Up until the 1990s, the benefit of treating isolated systolic hypertension (ISH) was an area of uncertainty. The trial was designed to examine if the treatment of systolic hypertension alone when diastolic BP is normal can be beneficial.

SHEP Study (22) Summary:

Design: double-blind Placebo controlled RCT
Inclusion: systolic BP > 160 mmHg, diastolic BP < 90 mmHg with age >60 years.
Total participants 4736; (active 2365, placebo 2371).

- Mean systolic at entry BP – 170, mean diastolic – 77
- Mean age – 72 (57% women)
- RX; chlorthalidone with a step-up to atenolol or reserpine if needed
  Step I – chlorthalidone 12.5 mg/day or Placebo
  Step II – CTD 25 mg/day or Placebo
  Step III – CTD 25 + Atenolol 25 mg/day or Placebo
  Step IV – CTD 25 + Atenolol 50 mg/day
  Step IV – CTD25 + Reserpine 0.05 mg/day
  K supplement PRN for K < 3.5 meq/l
- Average follow-up – 4.5 years
- Average BP at study end (control vs. active RX) – 155/72 vs. 143/65
- Incidence of stroke – 8.2 vs. 5.2% (RRR 36%)
- Incident of MI – 141 vs. 106 (RRR 23%)
- Incidence of CVD – 414 vs. 289 (RRR 22%)
- Deaths from all cause – 242 vs. 213 (RRR 13%)

Treatment of Mild Hypertension study (TOMHS) 1993 Randomized controlled trial that compared efficacy of 5 classes of antihypertensive drugs along with non-pharmacologic treatments

The TOMHS trial (23) was a randomized double-blind placebo controlled trial set up to compare the BP lowering effects of six treatment regimen in patients with stage 1 hypertension (defined as diastolic blood pressure DBP 90–99 mmHg and systolic blood pressure, SBP 140–159 mmHg).

The six treatment regimens were:

1. Placebo(nutritional-hygienic advice) *n* = 234
2. Chlorthalidone + nutritional-hygienic advice, *n* = 136
3. Acebutolol + nutritional-hygienic advice, *n* = 132
4. Doxazosin mesylate + nutritional-hygienic advice, *n* = 134
5. Amlodipine + nutritional-hygienic advice, *n* = 131
6. Enalapril + nutritional-hygienic advice, *n* = 135

Nutritional hygienic advice included increase in physical activity, weight reduction, lowering salt intake, and reducing number of alcoholic drinks.

Outcomes: all six groups had sizeable BP reductions with minimal differences between individual Drug intervention groups.

- Blood pressure: drug treatment vs. placebo (−15.9 vs. −9.1 mmHg for SBP and −12.3 vs. −8.6 mmHg for DBP).
- Death or non-fatal cardiovascular event: drug treatment vs. placebo (5.1 vs. 7.3%, *p* = 0.21).
- Other clinical events: drug treatment vs. placebo (11.1 vs. 16.2%, *p* = 0.03).

Based on the results, the investigators concluded that intervention with a drug and nutritional–hygienic advice was better compared to only nutritional–hygienic advice for treatment of mild hypertension.

Dietary Approaches to Stop Hypertension (DASH) 1997 Randomized controlled trial to determine if Mediterranean type diet with or without salt restriction lowers BP. This is one of the best prospective interventional diet studies in the hypertension research.

Up until the 1990s, the non-pharmacological efforts to reduce blood pressure relied on a mix of weight control, reduced salt intake, reduced alcohol intake, and to an extent increase in dietary potassium. Earlier studies examining the relationship between diet and blood pressure found small and inconsistent changes in BP by nutrients such as potassium, calcium, magnesium, protein, and fiber. But most of these studies were retrospective in nature based on diet recall. In 1994, the first phase of the prospective DASH study was initiated (24).

Design: multicenter, prospective, randomized controlled trial
Participants: 459, SBP < 160 mmHg, DBP between 80 and 95 mmHg, 133 hypertensive
Intervention: three sets of diets using prepared meal

- Control diet, comparable to the average American diet – potassium, calcium and magnesium at the 25th percentile of US consumption. Sodium 3 g.
- Rich in fruits and vegetables diet – potassium, calcium, magnesium at 75th percentile of US consumption with high fiber. Sodium 3 g.
- Combination diet – plenty of fruits and vegetables (9–10 servings/day), whole grain cereals, low fat dairy products and reduced saturated and total fat (Known now as DASH diet).
- All participants were initially fed control diet for 3 weeks (run in phase) followed by randomization for 8 weeks (intervention phase) into one of the diet groups.
  Results
    - In the hypertensive subset, combination diet reduced SBP and DBP by 11.4 and 5.5 mmHg, respectively, over the reduction by the control diet (*p* < 0.001).
    - In the non-hypertensive subset, combination diet reduced SBP and DBP by 5.5 and 3.0 mmHg, respectively, over the control diet.

Low Sodium DASH:

This part of the DASH trial evaluated the effect of lowering salt intake in combination with the DASH diet. Completed 4 years after the original DASH trial (2001), this part of the study had 412 participants randomized to DASH or control diet and within each of these 2 categories there were 3 levels of sodium intake: 3.5, 2.3, and 1.2 g daily.

Results:

High salt + control diet vs. Int salt + control-Sys BP lowered 2.1 mmHg
High salt + DASH diet vs. Int salt + DASH-Sys BP lowered 1.3 mmHg
Int slat + control diet vs. low salt + control-Sys BP lowered 4.6 mmHg
Int Salt + DASH diet vs. low salt + DASH Sys BP lowered 1.7 mmHg

There was additive BP lowering effect on already lowered BP with DASH diet with every lowered level of salt intake. Not surprisingly, benefit of BP lowering effect of low salt was more pronounced in the control diet. The best BP lowering was in the low salt DASH diet. Compared to the control with high salt diet DASH/low salt reduced BP by 7.1 and 11.5 mmHg in normotensive and hypertensive participants, respectively.

Medical research Council: Double-blind randomised trial of modest salt restriction in older people 1997 First study to explore effect of salt reduction in older people with normal range BP

At the time of the study, it was well known that high blood pressure could lead to stroke in older people. What was also known was that older people with normotensive blood pressure could also develop stroke. However, no efforts were made to lower the blood pressure in normotensive older people. The MRC study (25) aimed to answer just that question. The key highlights of the study are as below.

- Design: this double-blind randomized controlled crossover trial explored the effect that salt reduction would have on blood pressure in such individuals. The study participants included both normotensive and hypertensive individuals.
- In all 47 people were parts of the trial of which 18 were normotensive and 29 were hypertensive.
- In the normal salt intake for the UK population group, supine blood pressure was 163/90 (SD 21/10) mmHg with urinary sodium excretion of 177 (49) mmol/day.
- With modest sodium restriction, blood pressure fell to 156/87 (22/9) mmHg (*p* < 0.001) with a urinary sodium excretion of 94 (50) mmol/day.
- A reduction in sodium intake of 83 mmol/day was associated with a reduction of 7.2/3.2 mmHg. There was no significant difference in the blood pressure fall between 18 normotensive and 29 hypertensive participants (8.2/3.9 vs. 6.6/2.7 mmHg).

Hypertension Optimal Treatment (HOT) 1998 First large randomized control trial to determine if lowering target diastolic blood pressure below 90 mmHg reduces CV events further.

Since the second VA co-operative study, diastolic BP target had been 90 mmHg. No large trial was done since then to see if further lowering was beneficial. The Hypertension Optimal Treatment (26) (HOT) was a large multicenter trial designed to examine if lowering target diastolic pressure to 85 or 80 mmHg reduces CV events or mortality. A secondary objective of this study was to examine the effect of low dose aspirin in preventing stroke further persons with treated hypertension.

- Randomized control trial across 26 countries
- 18,790 patients in the age group of 50–80
- Diastolic blood pressure (DBP) between 100 and 1115 mmHg

- Three target Diastolic BP goal:
  ∘ DBP ≤ 90 mmHg (*n* = 6264)
  ∘ DBP ≤ 85 mmHg (*n* = 6264)
  ∘ DBP ≤ 80 mmHg (*n* = 6262)
- Meds used (same in all group): felodipine 5 mg daily to start and go up to 10 mg, add Ace inhibitor or beta blocker if target not achieved and finally add thiazide diuretic if still above target
- Additionally
  ∘ 9399 received acetylsalicylic acid
  ∘ 9391 received placebo
- Findings
  ∘ 20.3 mmHg reduction in DBP ≤ 90 mmHg group
  ∘ 22.3 mmHg reduction in DBP ≤ 85 mmHg group
  ∘ 24.3 mmHg reduction in DBP ≤ 80 mmHg group

The final result of the study failed to show any significant difference in outcome between all three groups. However, the actual achieved mean diastolic BP for the ≤90, ≤85, or ≤80 mmHg groups was 85, 83, and 81 mmHg, respectively. The difference in actual achieved diastolic BP degree of blood pressure reduction among the three groups was much smaller than the planned difference of 5 mmHg. So, the study failed to provide the power to detect any difference in protection with varying degrees of blood pressure lowering.

United Kingdom Prospective Diabetes Study (UKPDS) 1998 Largest and longest trial examining efficacy and outcome of different treatment modalities for T2DM one arm of which examined the treatment for hypertension in T2DM.

The United Kingdom Prospective Diabetes Study (27, 28) was set up to find answers for several key questions related to diabetes management. Two key questions that the study aimed to answer in relation to hypertension were (1) did tight control of blood pressure in diabetics have an effect on complications and (2) was there any specific advantage or disadvantage of using a beta blocker or angiotensin converting enzyme inhibitor in treating hypertension in this population.

Design: randomized control trial
Two groups:

- Tight blood pressure control (target < 150/85 mmHg), *n* = 758
- Less tight control group (target < 180/105 mmHg), *n* = 390

Results: tight control vs. light control

- Achieved Mean BP: 144/82 vs. 154/87 mmHg (*p* < 0.0001).

Reductions in risk of (Tight BP control vs. light BP control)

- Diabetes-related end points (microvascular complications) = 24% (95% CI 8–38%, *p* = 0.0046)
- Deaths related to diabetics = 32% (95% CI 6–51%, *p* = 0.019)
- Strokes = 44% (95%CI 11–65%, *p* = 0.013)
- Microvascular end points = 37% (95% CI 11–56%, *p* = 0.0092)
- Deterioration of retinopathy = 34% (99% CI 11–50%, *p* = 0.004)
- Deterioration in visual acuity = 47% (99% CI 7–70%, *p* = 0.004)

The study concluded that tight blood pressure control offered very significant benefits in various categories much more so than tight blood sugar control.

Of the 758 patients in tight BP control group 400 was allocated to treatment with captopril and 358 to atenolol. Mean achieved BP were 144/83 and 143/81 in captopril and atenolol groups, respectively. Captopril and atenolol were equally effective in reducing macro and microvascular endpoints.

African American Study of Kidney Disease (AASK) 2002 Landmark study in African Americans for determining target blood pressure and suitable drug regimen in hypertension control to prevent progressive renal failure. The study failed to show benefits of tight BP control over usual control to slow decline in GFR.

Hypertension is the second leading cause of End Stage Renal Disease (ESRD) and African-Americans are six times likelier to progress to ESRD from hypertension as compared to whites (29). The African-American Study of Kidney Disease published in 2002 (21), compared the effects of two levels of blood pressure control and three drug regimens on the rate of decline in glomerular filtration rate(GFR) in African-Americans with hypertension and chronic kidney disease (CKD).

Design: 2 × 2 factorial design Randomized control trial.
Inclusion criteria:
  - African-Americans
  - 18–70 years of age
  - Hypertensive renal disease (GFR, 20–65 ml/min per 1.73 m^2^)
Intervention groups
Goal BP:
  Gr. A. Lower mean arterial pressure group (target BP 92 mmHg or less), *n* = 540
  Gr. B Usual mean arterial pressure groups (BP 102–107 mmHg), *n* = 554
  Each group then was randomized to three different classes of antihypertensive agents
  - Beta blocker (metoprolol), *n* = 441
  - Angiotensin converting enzyme inhibitor (Ramipril), *n* = 436
  - Dihydropyridine calcium channel blocker (Amlodipine), *n* = 217

All patients could use diuretics, direct vasodilator, alpha blocker, and central sympatholyitics as needed (in addition to lifestyle changes to lower salt intakes, etc.) to reach goal BP.

Outcomes:
Lower target BP group vs. usual BP group
  - Achieved BP
    ∘ 128/78 mmHg, SD (12/8 mmHg) vs. 141/85 mmHg, SD (12/7 mmHg)
  - Mean (SE) GFR slope
    ∘ 1-2.21 (0.17) ml/min per 1.73 m^2^/year vs. −1.95 (0.17) ml/min per 1.73m^2^ per year (*p* = 0.24)
  - Composite clinical outcomes risk reduction for lower blood pressure group
    ∘ 2% (95% CI −22 to 21%; *p* = 0.85)
Drug comparison
  - Risk reduction in composite clinical outcome
    ∘ Ramipril vs. metoprolol, 22% (95% CI 1–38%, *p* = 0.04)
    ∘ Ramipril vs. amlodipine, 38% (95% CI 14–56%, *p* = 0.004)

The authors observed that there was no significant slowing of disease progression (hypertensive nephrosclerosis) with a lower target BP. However, the results suggested that Ramipril was more effective compared to amlodipine in slowing decline in GFR. Superiority of Ramipril over Metoprolol was just marginal.

Antihypertensive and Lipid Lowering Treatment to Prevent Heart Attack Trial (ALLHAT) 2002 Largest antihypertensive trial. Resolved the question of superiority of any of the newer class of antihypertensive drugs over the old thiazide like diuretic Chlorthalidone.

This large trial involving about 42,000 participants were designed to answer the important and lingering question whether newer classes of drugs such as angiotensin converting enzyme inhibitor (ACEI), calcium channel blocker (CCB), and alpha blocker give better CV outcomes than the older drugs such as thiazide diuretics (30).

Design: double-blind randomized control trial
Participants: patients, aged over 55 years with treated or untreated hypertension, with one additional Coronary heart disease risk factor.
The participants were from USA and Canada with 35% participants from AA community.
Four groups:
Chlorthalidone chosen drug representing thiazide/thiazide-like diuretics.
Total participants (15,525, higher than other groups since each of the other was being compared to this group);
Amlodipine represented CCB (*n* = 9048);
Lisinopril represented ACEI (*n* = 9054);
Doxazosin represented alpha blocker (*n* ~ 9061).
Outcome measures: the Doxazosin arm was terminated early when interim analysis by safety monitoring board concluded that there was a very high incidence of congestive heart failure in this arm. Outcome analyses on all the primary and secondary outcomes were done on the remaining three arms.
Results:
  - Primary – fatal CHD or non-fatal MI
  - Secondary – all cause mortality, stroke, combined CHD, combined CVD
Main results:
Primary outcomes: *n* = 2956; Chlorthalidone (11.5%); Amlodipine (11.3%), Lisinopril (11.4%);
Compared with Chlorthalidone, RR of Amlodipine was 0.98 (95% CI 0.90–1.07);
Compared with Chlorthalidone RR of Lisinopril was 0.99 (95% CI 0.91–1.08).
Key Secondary outcomes
  - Amlodipine vs. Chlorthalidone for heart failure (10.2 vs. 7.7%; RR 1.38; 95% CI 1.25–1.52)
  - Lisinopril vs. Chlorthalidone
    ∘ For CVD (33.3 vs. 30.9%; RR 1.10; 95% CI 1.05–1.16)
    ∘ For stroke (6.3 vs. 5.6%; RR 1.15; 95% CI 1.02–1.30)
    ∘ For Heart Failure (8.7 vs. 7.7%; RR, 1.19; 95% CI 1.07–1.31)

Based on these findings, ALLHAT study authors concluded that thiazide should be the preferred antihypertensive to start with unless there is clear contraindication. However, it was an optimistic generalization about thiazide as a group and future studies and analysis showed that all thiazides are not similarly effective and the benefits of Chlorthalidone as antihypertensive is not equaled by commonly used hydrochlorothiazide.

Australian National Blood Pressure Study (ANBP II) 2003 Contemporary to ALLHAT study, done in a different population but with same goal of comparison of thiazide vs. Ace inhibitor as antihypertensive drug

The results of the Second Australian National Blood Pressure Study (ANBP2) followed soon after ALLHAT results were published. This was a randomized, open-label, blinded end point study of 6083 hypertensive patients aged 65–84 years, who otherwise had a relatively low cardiovascular risk profile, with a median follow-up of 4.1 years (31). Initial treatment options included ACE-I or a diuretics-based regimen (HCTZ and Enalapril were the recommended agents). The key findings from the study were:

Intervention: enalapril vs. HCTZ

- All CV events or death from any cause
  – HR = 0.89 (0.79–1.00), *p* = 0.05
  – CVD: HR = 0.88 (0.77–1.01), *p* = 0.07
  – CHD: HR = 0.86 (0.70–1.06), *p* = 0.16
  – Stroke: HR = 1.02 (0.78–1.33), *p* = 0.91
  – HF: HR = 0.85 (0.62–1.18), *p* = 0.33

Anglo-Scandinavian Cardiac Outcomes Trial-Blood Pressure Lowering Arm ASCOT BPLA 2005 After the comparison of individual antihypertensive trials, next series of trials focused on the outcome with different combinations of antihypertensive drugs. ASCOT was one such early trial comparing CCB and ACEI with Beta blocker and thiazide diuretic

Published in 2005 the Anglo-Scandinavian Cardiac Outcomes Trial-Blood Pressure Lowering Arm (ASCOT-BPLA) (32) was a randomized, open-label, blinded end point, controlled study. The main aim of the study was to compare two treatment regimens; atenolol and a thiazide (representing the older drugs) vs. amlodipine and perindopril (the newer classes).

Design: international multi-center randomized control trial
Participants: total 19,257 hypertensives male and female in the age group of 40–79 years, having at least one additional cardiovascular risk factors.
Two regimens
  - Amlodipine ± perindopril (*n* = 9639) vs. Atenolol ± bendroflumethiazide (*n* = 9618)
Primary end point: non-fatal MI and fatal CHD
Outcomes: amlodipine regimen vs. atenolol regimen
Primary end point – (429 vs. 474; unadjusted HR 0.90, 95% CI 0.79–1.02, *p* = 0.1052)
Fatal and non-fatal stroke – (327 vs. 422; HR, 0.77, 95% CI 0.66–0.89, *p* = 0.0003)
Total cardio-vascular events and procedures – (1362 vs.1602, HR,0.84, 95% CI 0.78–0.90, *p* < 0.0001)
All-cause mortality – (738 vs. 820, HR 0.89, 95% CI 0.81–0.99, *p* = 0.025)
Incidence of diabetes – (567 vs. 799, HR 0.70, 95% CI 0.63–0.78, *p* < 0.0001)

The results suggested that use of a CCB with addition of ACEI gives much better outcome than older regimen of beta blocker and thiazide.

Of note, this study like ANBP was open label trial (unlike ALLHAT) and used hydrochlorothiazide representing the thiazide class (not Chlorthalidone as in ALLHAT and other NIH sponsored studies). These crucial differences possibly account for different result in ANBP compared to ALLHAT.

Conduit Artery Function Evaluation (CAFE) 2006 This is a substudy of the ASCOT and this is the only large study that examined the variable effect of different class of drugs on reducing central aortic pressure (as measured by the new non-invasive technique of applanation tonometry) vs. usual peripheral blood pressures(brachial pressure). This study gave answer to the lingering question why beta blocker like atenolol was not very effective in reducing stroke in hypertensive patients.

The CAFE study (33) was a large sub study of the ASCOT trial. The CAFE trial was primarily designed to investigate differences in central aortic pressures vs. cuff BP in the two groups in ASCOT participants. The secondary aim of the trial was to examine if a relationship existed between central aortic pressures and cardiovascular outcomes. The central aortic BP was measured by use of applanation tonometry (Sphygmocore).

Total participants 2199.
Atenolol based (*n* = 1031)
Amlodipine based (*n* = 1042)
Baseline seated BP for two groups:
Atenolol based: 159.9/92.4 mmHg
Amlodipine based: 161/92.6 mmHg
Outcomes:
  - Brachial BP lowering:
    ∘ Atenolol: down to 133.9/78.6 (lowered −26/−13.8 mmHg)
    ∘ Amlodipine Down to 133.2/76.9 (−27.8/−15.7 mmHg)
  - Difference between two groups: 0.7/1.6 (*p* = 0.2)
  - Central BP
    ∘ Atenolol: 125.5/79 mmHg
    ∘ Amlodipine: 121.2/77.8 mmHg
    ∘ Difference between two groups: 4.3/1.4 (*p* < 0.0001)

The authors concluded that different BP lowering drugs can have different effects on the central aortic pressure and that could possibly explain the difference in outcomes of different anti-hypertensive drugs.

Hypertension in the Very Elderly Trial (HYVET) 2008 First randomized trial to demonstrate benefits of treating hypertension in very elderly

Prior to the HYVET (34) trial, there were conflicting views on treating very old patients with hypertension. There were some studies which suggested that blood pressure and death were inversely related, possibly because of adverse effects of treatment. Some studies suggested decreased strokes and heart failure amongst treated older populations while many studies enrolled very few older population, so as not to be able to derive any significant conclusion. The HYVET trial aimed to address these issues and enrolled 3845 patients across Australasia, China, Europe, and Tunisia. The results from the trial summarized below suggest that treatment of hypertension in the very elderly clearly is beneficial on all accounts.

Design: randomized control trial
Inclusion criteria:
  - 80 years or older
  - Systolic blood pressure > 160 mmHg
Regimens:
  - Active treatment (indapamide with or without perindopril) *n* = 1933
  - Placebo group, *n* = 1912
Outcomes:
  - BP in the active treatment group was 15/6.1 mmHg lower than the placebo group
In the intent to treat analysis, the active treatment group had
  - 30% reduction in the rate of fatal and non-fatal stroke (95% CI −1 to 51, *p* = 0.06)
  - 39% reduction in rate of death from stroke (95% CI 1–62, *p* = 0.05)
  - 21% reduction in rate of death from any causes (95% CI 4–35, *p* = 0.02)
  - 23% reduction in the rate of death from cardiovascular causes (95% CI −1 to 40, *p* = 0.06)
  - 64% reduction in the rate of heart failure (95% CI 42–78, *p* < 0.001)
  - Side effects: active treatment vs. placebo (358 vs. 448, *p* = 0.001)

Avoiding Cardiovascular Events through Combination Therapy in Patients Living with Systolic Hypertension (ACCOMPLISH) 2008 Large randomized trial that compared two commonly used treatment regimens (ACE + CCB vs. ACE + diuretic)

The Avoiding Cardiovascular Events through Combination Therapy in Patients Living with Systolic Hypertension (ACCOMPLISH) trial (35) explored the hypothesis that treatment of hypertensive patients, at a risk of cardiovascular disease was better with a combination of an angiotensin converting enzyme inhibitor (benazepril) and a calcium channel blocker (amlodipine) than a combination of an ACE inhibitor (benazepril) and a diuretic (hydrochlorothiazide) as recommended by then prevailing JNC 7 guidelines. The key findings of the study are as follows:

Design: double-blind, randomized
Participants: *n* = 11,506 patients with hypertension with at least one additional CV risk factors
Regimens: Benazepril + Amlodipine (*n* = 5744) vs. Benazepril + Hydrochlorothiazide (*n* = 5762)
Mean blood pressure after dose adjustment
  - Benazepril + Amlodipine = 131.6/73.3 mmHg
  - Benazepril + Hydrochlorothiazide = 132.5/74.4 mmHg

Outcome events: (a composite of death from cardiovascular causes, non-fatal myocardial infarction, non-fatal stroke, hospitalization for angina, resuscitation after sudden cardiac death, or coronary revascularization)

- Benazepril + Amlodipine = 552 (9.6%)
- Benazepril + Hydrochlorothiazide = 679 (11.8%)
  2.2% absolute risk reduction with Benazepril + Amlodipine
  19.6% relative risk reduction with Benazepril + Amlodipine (hazard ratio, 0.80, 95% CI 0.72–0.90, *p* < 0.001).

At the end of the study, which was stopped prematurely after attaining a pre-determined end point, the authors concluded that the ACEI and CCB combination was superior to the ACEI and diuretic combination in reducing cardiovascular events in patients with hypertension at risk for CV events.

The two major studies, ASCOT-BPLA and ACCOMPLISH, compared the newer and popular regimens (CCB and ACEI) vs. older drugs such as beta blockers and thiazide diuretics. Both were very large studies, but both were open label and sponsored by pharmaceutical companies. One of the major weaknesses in accepting the conclusion of thiazide being less effective than CCB when in combination with another class is that these studies invariably used a weak thiazide diuretic like hydrochlorothiazide. All NIH studies used Chlorthalidone and not hydrochlo­rothiazide as the diuretic. A recent network meta-analysis had confirmed that Chlorthalidone is much superior to hydrochlorothiazide in efficacy and cardiovascular outcomes. Why these two studies used hydrochlorothiazide when a superior drug, Chlorthalidone was available is open to speculation.

Action to Control Cardiovascular Risk in Diabetes (ACCORD) 2010 Until ACCORD studies, prevailing wisdom was in diabetic lower the BP is better but this notion was not evidence based. ACCORD trial was designed to answer this very important question.

The Joint National Committee on Prevention, Detection, Evaluation and Treatment of High Blood Pressure (JNC 7) recommended lowering systolic blood pressure to <130 mmHg in diabetics (30). The Action to Control Cardiovascular Risk in Diabetes (ACCORD) trial (36) tested the effect of lowering the systolic blood pressure to <120 mmHg on cardiovascular events in diabetic patients compared to the usual systolic 140. The primary findings of the study were as follows:

Design: double-blind randomized control trial
No of participants # 4733
  - Intensive therapy (IT, target systolic BP < 120 mmHg) *n* = 2362
  - Standard therapy (ST, target systolic BP < 140 mmHg) *n* = 2371

Primary outcome = non-fatal MI, non-fatal stroke, or death from any cardiovascular causes

At the end of study with mean follow-up of 4.7 years:

Mean systolic BP: (IT) 119.3 mmHg vs. (ST) 133.5 mmHg
Primary outcome rate: 1.87% (IT) vs. 2.09% (ST) (hazard ratio, 0.59; 95% CI 0.73–1.06, *p* = 0.20)
Annual rate of death (any cause): 1.28% (IT) vs. 1.19% (ST) (HR, 1.07; 95% CI 0.85–1.35, *p* = 0.55)
Annual rate of stroke: 0.32% (IT) vs. 0.53% (ST) (HR 0.59; 95% CI 0.39–0.89; *p* = 0.01)
Serious adverse events of anti HT therapy: IT – 77 (3.3%) vs. ST-30 (1.3%) (*p* < 0.001)

The authors concluded that compared to usual BP control intensive BP control in diabetics does not confer any significant benefit.

Primary Prevention of Cardiovascular Disease with a Mediterranean Diet (PREDIMED) 2013 First randomized control trial that studied the effect of a Mediterranean diet on Cardiovascular Disease

This was a large multicenter study (37) in Spain with over 7400 participants enrolled in the study. There were three main groups. The first group was advised a diet with extra virgin olive oil, the second group with Mediterranean nuts, and the third with the control group.

Total participants = 7747
Age of participants = 55–80 years, 57% women
Median follow-up = 4.8 years
Primary cardio vascular end point occurred in 288 participants
Extra virgin olive oil = 96 events, adjusted Hazard ratio 0.70 (95% CI 0.54–0.92)
Mediterranean nuts = 83 events, adjusted Hazard ratio 0.72 (95% CI 0.54–0.96)
Control group = 109 events

Systolic hypertension intervention trial (SPRINT) 2015 The SPRINT study conclusively demonstrated the benefit of lowering systolic blood pressure goal in a non-diabetic population.

The SPRINT (38) study was someway similar to ACCORD study but was in non-diabetics and without any prior history of stroke. It enrolled about 9000 participants, many of them elderly and with stage 2–4 chronic kidney disease. The study was stopped in the first week of September 2015 per recommendation of the DSMB due to huge reduction in mortality (25%) and CV events (30%) in the group with systolic BP goal of 120.

Total participants = 9361
Intensive Blood Pressure control group (systolic < 120 mmHg) = 4678
Standard Blood Pressure control group (systolic < 140 mmHg) = 4683
Primary end point was myocardial infarction, acute coronary syndrome, stroke, heart failure, or death from cardiovascular events.
At the end of 1 year systolic blood pressure achieved was
121.4 mmHg in the intensive treatment group
136.2 mmHg in standard control group
Median follow-up = 3.26 years
Primary composite outcome = (1.65 vs. 2.19% per year) intensive vs. standard, respectively. Hazard ratio in the intensive treatment group was 0.75; 95% confidence interval (CI), 0.64–0.89; (*p* < 0.001).

All cause mortality was found to be lower in the intensive treatment group: (hazard ratio, 0.73; 95% CI 0.60–0.90; *p* = 0.003).

## Conclusion

Hypertension is still the most prevalent of all non-­communicable chronic disease (NCCD) throughout the world including less developed countries. Per NHNES survey of 2011–2012, the prevalence of hypertension in the US has remained around 30% – and while awareness of hypertension has reached a laudatory goal of 83% – the control rate is still only 50% (49% for men and 55% for women). These rates are also very similar among all ethnic groups. Therefore, though we have come a long way in the last 50 years in managing hypertension – from calling it essential to finding effective drug therapy for control – we are still short of truly overcoming this common malady of humanity. In fact, the annual number of deaths from HTN in the US still exceeds 30,000. Moreover, our understanding of the pathophysiology of common or so-called “essential” hypertension is still incomplete in many ways. The new discovery of extra-renal deposition of excess sodium in the skin, modulated by macrophages, changed the paradigm of classical Guytonian frame work of salt and hypertension (39, 40). Similarly, the role of immunity, especially the central indispensable role of T-cell infiltration in the kidney for Angiotensin II-mediated hypertension also made the pathophysiology of hypertension more complex (41, 42). Of course, there is the evolving understanding of the tremendous role played by the trillions of gut microbiota in health and disease overall, including hypertension (43). These are only a few of the many new developments in broadening our understanding of the pathophysiology of hypertension. Without understanding the disease (or possibly the complex of diseases that manifest as high BP), we cannot achieve true control over it.

We know many isolated small communities like Kuna Indians, who continue to follow their ancestral traditions, do not develop hypertension even in their very old age. It is possibly not gene-related, since they develop hypertension when they immigrate to western communities (44, 45). Thus, we have to continue to strive to expand our knowledge of the physiology and epidemiology of hypertension and related diseases. We must explore other potential non-drug therapies. Renal sympathetic nerve ablation showed some initial promise but needs lots of work to determine its role (46). Similarly, the role of different lifestyle improvements including optimal salt and fruits/vegetable intake, role of flavonoids from cocoa, coffee, or tea needs further exploration and refinement.

Regarding immediate clinic management of hypertension, a major unresolved issue is to define the exact clinical utility and wide applicability of ambulatory BP monitoring as well as the role of home BP monitoring. We know that the 24-h average BP per ABPM predicts hypertension outcome better than office BP – the standard used in almost all the trials cited above. We also know for the same level of office BP, people with non-dipping night BP has worse prognosis in CV outcomes (47). Wider use of ABPM can further reduce the high incidence of stroke and heart failure in the overall population.

There are many other worthy issues related to hypertension that we cannot adequately discuss in this space. We can only say that the great success in refining hypertension treatment, so far in the last 50 years is truly only the beginning of a long journey. The challenge remains for the next generation of clinicians and researchers for further advancement as we better understand human biology.

The success of pharmacological treatment of a common human malady like hypertension that affects more than a billion of our fellow human beings is a mega achievement. In the history of modern medicine, only vaccination to prevent infectious diseases, antibiotics for infections, and oral hydration for diarrheal diseases have had similar success and impact on global health.

In some ways, the success of treating this common chronic non-communicable disease (NCD) – as WHO has dubbed it – made way for effective planning to manage others. The success of multi-center RCTs for hypertension led to the concept of a very large multi-center study using one single variable intervention (aspirin for preventing CAD or ACE I for systolic heart failure) (48). In many ways, this eventually led to the modern concept of Evidence Based Medicine (EBM), which is now the cornerstone of scientific medicine.

In my experience in teaching medical students and residents, I noticed how little they know about these major breakthroughs in medicine especially cardiovascular medicine in the last quarter of the twentieth century and how the success of pharmacologic treatment of hypertension started this revolution. Once they learned about it, they got a better perspective and better understanding of the evolving nature of the Evidence-Based Medicine. It is these trainees that I had in mind when I decided to create this timeline of the history of hypertension treatment. I hope that the next generation will carry on the spirit of learning and conquer the uncharted territory of this common malady that affects a billion men and women worldwide today.

## Author Contributions

MS planned the review, did the initial literature search in addition to what was already known to him and selected studies to be included. He also wrote the introduction and the conclusion. Most of the short comments on individual studies were written by him. He also selected many of the references that are included. ND helped gathering details of data from selected studies and summarizing them. He also helped creating the figure.

## Conflict of Interest Statement

The authors declare that the research was conducted in the absence of any commercial or financial relationships that could be construed as a potential conflict of interest.

## Abbreviations

BP, blood pressure; HTN, hypertension; Dias, Diastolic; Sys, systolic; CV, cardiovascular; CVD, cardiovascular death; MI, myocardial infarction; CKD, chronic kidney disease; ARR, absolute risk reduction; RR, relative risk reduction. Drugs: CCB, calcium channel blocker; BB, beta blocker; ACEI, angiotensin converting enzyme inhibitor; HCTZ, hydrochlorothiazide; CTDN, chlorthalidone; AB, alpha blocker; ARB, angiotensin receptor blocker. Trials: VACoop, Veterans Affairs Co-operative studies; HDFP, hypertension detection and follow-up trial; MRFIT, multiple risk factor intervention trial; MRC, Medical Research Council (UK); EWHPE, European Working Party High Blood pressure in the Elderly; SHEP, systolic hypertension in the elderly program; TOMHS, treatment of mild hypertension study; DASH, dietary approaches to stop hypertension; HOT, hypertension optimal treatment; UKPDS, United Kingdom Prospective Diabetes Study; AASK, African-American Study of Kidney Disease; ALLHAT, antihypertensive and lipid lowering treatment to prevent heart attack trial; ANBP, Australian National Blood Pressure Study; ASCOT-BPLA, Anglo-Scandinavian Cardiac Outcomes Trial-Blood Pressure Lowering Arm; CAFÉ, Conduit Artery Function Evaluation; HYVET, hypertension in the very elderly trial; ACCOMPLISH, avoiding cardiovascular events through combination therapy in patients living with systolic hypertension; ACCORD, Action to Control Cardiovascular Risk in Diabetes; SPRINT, systolic blood pressure intervention trial.
